# Long-Term Follow-Up before and during Riluzole Treatment in Six Patients from Two Families with Spinocerebellar Ataxia Type 7

**DOI:** 10.1007/s12311-024-01714-w

**Published:** 2024-07-08

**Authors:** Agnese Suppiej, Chiara Ceccato, Radouil Tzekov, Iveta Cermakova, Francesco Parmeggiani, Gianmarco Bellucci, Marco Salvetti, Theresa Zesiewicz, Giovanni Ristori, Silvia Romano

**Affiliations:** 1 Department of Medical Sciences, University of Ferrara, Ferrara, Italy; 2 Robert Hollman Foundation, Padova, Italy; 3 ERN-EYE Network - Center for Retinitis Pigmentosa of Veneto Region, Camposampiero Hospital, Camposampiero (Padova), Italy; 4 Department of Ophthalmology, University of South Florida, Tampa, FL USA; 5 Department of Translational Medicine for Romagna, University of Ferrara, Ferrara, Italy; 6 Center for Experimental Neurological Therapies, Department of Neurosciences, Mental Health and Sensory Organs (NESMOS), Faculty of Medicine and Psychology, Sant’Andrea Hospital, Sapienza University of Rome, Rome, Italy; 7 IRCCS Istituto Neurologico Mediterraneo (INM) Neuromed (M.S.), Pozzilli, IS Italy; 8 Neuroimmunology Unit, Fondazione Santa Lucia, Rome, Italy; 9 Department of Neurology, University of South Florida, Tampa, FL USA

**Keywords:** Spinocerebellar degenerations, Cerebellar ataxia, Inherited retinal dystrophies, Paediatric, Natural history

## Abstract

**Background:**

Currently no curative treatment exists for spinocerebellar ataxias (SCAs). Riluzole repurposing was proposed as a symptomatic treatment in different types of cerebellar ataxia. We report a long-term-follow up under riluzole treatment in SCA type 7.

**Methods:**

Six patients received Riluzole 50 mg twice daily on a compassionate use program for a mean of 4.8 years (range 3.5-9). We measured ataxia onset and progression through the Scale for the Assessment and Rating of Ataxia (SARA), and collected extensive ophthalmological data before and after Riluzole treatment. Electrocardiogram and laboratory profile for drug safety were performed every six months.

**Results:**

Riluzole treatment showed no effect on visual function in two patients with an advanced retinal damage. Improvements of visual function occurred in four patients followed by ophthalmologic stability up to 5 years after starting treatment. Two patients had a less steep deterioration of ataxia after treatment compared to pre-treatment, during the first 2,5 years of therapy. One showed soon after therapy an improvement of the SARA score, and then overall stability lasting 3,5 years, followed by ataxia worsening. One visually impaired patient without neurological impairment did not worse until the last visit after 3,5 years of follow-up. The remaining 2 patients showed an improvement of SARA scores soon after therapy, and an overall stability lasting respectively 5 and 3 years. No adverse event was registered during the observation period.

**Discussion:**

This study suggests a possible beneficial action of Riluzole in SCA7 and provides a detailed description of the ophthalmologic profile of these patients.

## Introduction

Spinocerebellar ataxia type 7 (SCA 7) is a genetically determined neurodegenerative disorder characterized by progressive neurological manifestations and visual loss that highly impact quality of life. It belongs to the autosomal dominant cerebellar ataxia family (ADCAs) being one of the rarest accounting for 1 to 11.7% of genetically diagnosed ADCAs [1].

The disease is caused by abnormal CAG repeat expansions in the ATXN7 gene, located on chromosome 3, and alters ataxin-7 protein function, an 892 amino acid protein widely expressed in human tissue, including the retina. During meiosis CAG repeat number tends to expand (37–306 CAG repeats near the N-terminus) due to a strong anticipation phenomenon showing one of the highest tendencies among heritable unstable trinucleotide repeats disorders [2, 3], with large expansions more frequently transmitted by father rather than mothers [4–6]. Repeats length correlates inversely with the age at onset and directly with the disease severity: larger expansions determine earlier onset and more severe manifestations.

Visual loss in SCA 7 is primary due to cone-rod photoreceptor degeneration that causes visual acuity deterioration in the 83% of patients [7]. However, the retinal phenotype has not been fully elucidated. Being able to suspect SCA7 by retinal degeneration could be of high interest since visual symptoms may precede neurological involvement in juvenile onset forms [4, 8, 9].

Current SCA therapeutic strategies include gene suppression to silence disease-causing mutant protein expression and targeting convergent mechanisms of disease [10]. As a symptomatic approach, riluzole, a potassium channel activator in use for treatment of amyotrophic lateral sclerosis, seems promising in a mixed population of SCAs without retinal involvement [11], although a recent study failed to confirm this effect in patients with spinocerebellar ataxia type 2 [12]. The mechanism of action of riluzole in SCAs is not fully understood, but the action of small-conductance potassium channel openers is a plausible working hypothesis [13–15]. Additional pleiotropic neuroprotective effects were described: the drug enhances the uptake of glutamate by astrocytes and reduces the release of glutamate from active synapses, counteracting damage of excitotoxicity; it also enhances activity of the TWIK-related potassium channel-1 (TREK-1) and intracellular expression of heat shock proteins that have a neuroprotective role and counteract blood-brain barrier dysfunction [16, 17]. TREK channels activity also seems to play a role in inhibiting retinal cell apoptosis. Shen et al. observed that riluzole could protect human retinal pigment epithelium (RPE) cells against oxidative injury-induced cell death [18–23].

To our knowledge, there are no available clinical studies that investigate the use of riluzole on SCA 7, where a possible effectiveness could be observed not only on the neurological disorder but also on the retina dysfunction.

The present study is aimed at describing the clinical characteristics at onset and the long-term follow-up of a two generations family living in Italy and of two siblings living in the USA with genetic diagnosis of SCA 7. All have been treated with riluzole on a compassionate use program. The focus is on early visual symptoms, the retinal phenotype and the evolution of visual and neurologic symptoms before and during riluzole treatment.

## Methods

The study cohort includes five patients of Peruvian origin belonging to the same family recruited at the Robert Hollman Foundation (RHF, Italy), and two American sisters they were recruited for neurological evaluation and treatment at the Ataxia Research Center at the University of South Florida and for ophthalmic evaluation at the Department of Ophthalmology at the University of South Florida. In all, genomic DNA isolated from blood and analysed by a polymerase chain reaction assay confirmed SCA7 diagnosis. Early clinical data was based on information from all available ophthalmological and neurological records. The age at onset was defined as the age at which the patient first noticed the symptom, irrespective of whether a medical contact was made or not at that time. The data from anamnestic interviews were analysed with respect to the first visual symptoms or first neurological complaints. This was done in order to collect information about which symptoms occurred first. Clinical data was collected through family history, medical reports and prospectively from 2017 to December 2020 at RHF and from 2006 to 2020 at the Ataxia Research Center and the Department of Opthalmology and at the University of South Florida.

All patients were periodically monitored at baseline and during assumption of riluzole on a compassionate use program. Data on ataxia-associated symptoms using the Scale for the Assessment and Rating of Ataxia (SARA) and on visual function primarily testing visual acuity (VA) with logarithmic Snellen chart (logMAR notation used) were collected. Contrast sensitivity with Vistech electronic optotype, colour sensitivity with Ishihara pseudoisocromatic colour plates and colour vision test Roth 28, computerized threshold 30° visual field testing, full-field ERG, multi-focal ERG, visual evoked potential by flash and pattern-reversal stimuli, ocular motility and fixation were as well collected when performed during ophthalmological assessment. Macular optical coherence tomography (OCT) and fundus photography was periodically performed as part of the ophthalmological imaging follow-up session.

**Fig. 1:**
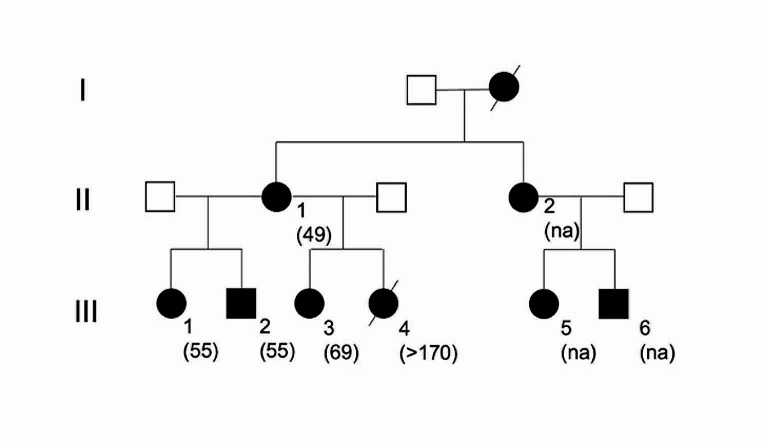
Family pedigree. Circle symbol, women; square: male; filled symbol, affected; unfilled symbol, unaffected; slashed symbol, deceased member. In brackets: number of CAG repeats

Electrocardiogram and laboratory profile (cell blood count, liver enzymes including AST, ALT, GGT, bilirubin and creatinine) for drug safety were performed every three months. Patient III-4 (see Fig. 1) did not receive riluzole treatment because of premature death.

## Results

### Medical Histories

The Peruvian family had five affected members, 4 female and 1 male. Figure 1 shows the family pedigree and number of CAG repeats: PII-1: 49, PIII-1:55, PIII-2:55, PIII-3:63, PIII-4: >170. Family history highlighted in the maternal line one affected woman with ataxia and low vision in adult age clinically diagnosed in Peru (PI) died because of throat cancer at the age of 59 and other three family members (PII-2, PIII-5, PIII-6) with vision impairment without neurological symptoms living in Peru. None performed genetic testing and clinical data were not available. American siblings had no available family history and have respectively S1:39 and S2:40 number of CAG repeats.

Patients PII-1, PIII-1, PIII-2, PIII-3 (CAG 49–63 range) presented as first symptoms visual loss and light sensitivity not associated to any neurological signs at a median age of 11,5 years (8–14 years range). S1 (CAG 39) had some visual symptoms reported like blurred vision before any overt neurologic symptomatology at 63 years. S2 (CAG 40) reported motor imbalance since the age of 48 years preceded by visual symptoms; at the age of 53 years, she was already found with mild visual impairment at the first available ophthalmological examination. Ataxia showed up at 35, 17, 10.5, 66, 48 years respectively in patients PII-1, PIII-1, PIII-3, S1, S2 whereas it was still not present in patient PIII-2 at the last follow-up at the age of 23 years. Patients PII-1, PIII-1, PIII-2, PIII-3, S1, S2 started riluzole therapy, respectively, at age 40.5, 21.5, 20, 11.5, 68, 57 years. Routine laboratory tests (including hepatic function) and electrocardiograms were normal at baseline and persisted normal at follow-up. All of them are alive at last follow-up at the age of 45, 25, 23, 15, 73 and 66 years.

In patient PIII-4, with a highly expanded CAG repeat greater than 170, the onset was at the age of sixteen months, with psychomotor development regression and ataxia followed after 6 months by visual impairment. She died at the age of 4 years.

### Visual Function Results

**Fig. 2:**
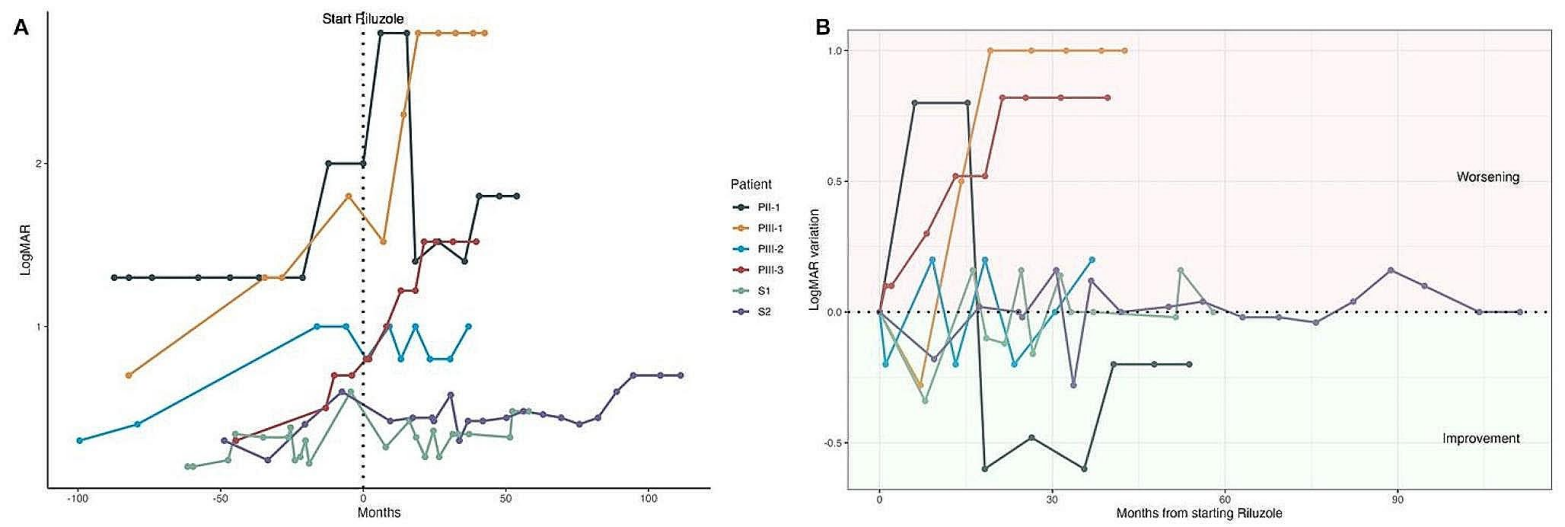
**A**: Visual acuity deterioration trend before and after riluzole treatment equalized at therapy onset (dotted vertical line); **B**: Visual acuity variation trend during riluzole treatment

Visual acuity loss at first ophthalmological testing ranged from 0.2 to 0.7 logMAR and was associated with normal contrast sensitivity, color vision, ocular motility and fixation. Vision progressively deteriorated from onset to the last ophthalmological follow-up showing different patterns of visual function loss according to combination of factors such as CAG repeat number and disease stage at therapy baseline. Figure 2 summarizes visual acuity trend before and after riluzole treatment in all alive patients.

Visual acuity impairment in all cases was followed by contrast and color sensitivity deficits, eccentric fixation and hypometric saccadic and smooth pursuit movements till the development over time of complete ophthalmoplegia in patients with blindness category 3 and 4 (PII-1, PIII-1, PIII-3), according to the Recommendations of the WHO Consultation on “Development of Standards for Characterization of Vision Loss and Visual Functioning” (2003). At the last follow-up visual acuity ranged from 0.48 logMAR to light perception (2.8 logMAR), all showed lower visual acuities than baseline except PIII-2 who maintained an overall stability of visual acuity equal to 1.0 logMAR even if he developed a red-green color defect. Table 1 shows the visual function values and their evolution from onset to the last follow-up.

Patient PIII-4 (CAG > 170) only evaluated after the neurological onset, at the age twenty-nine months showed resolution visual acuity under the age norms; no other data on visual functions was available thereafter, she was described as behaviorally blind during the last year of life.

**Table 1:**
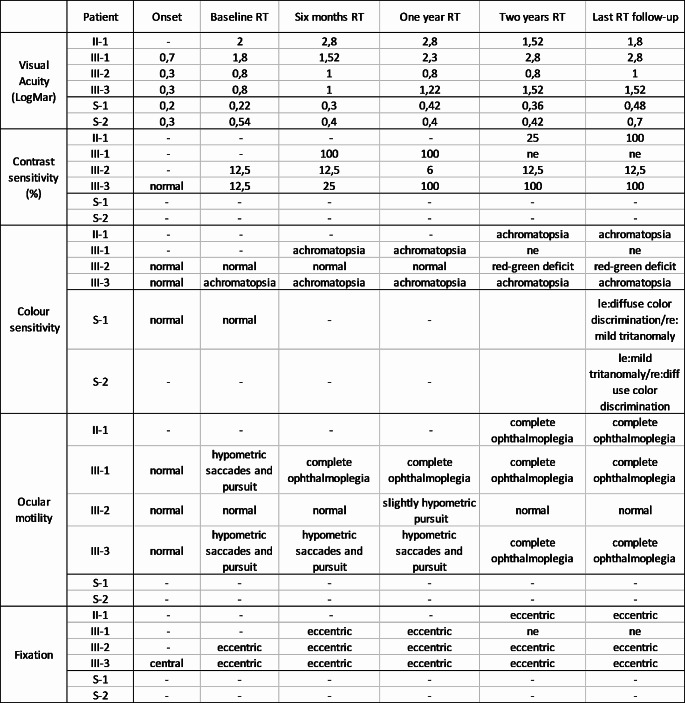
Visual function values from onset to baseline and last follow-up. RT: riluzole treatment; - not available data; ne: not evaluable

### Visual Electrophysiology

No ERG and VEPs data were available before treatment and at baseline for S1 and S2.

Early electrophysiological testing close to visual onset showed abnormal function of macular/paramacular cones in PIII-2 and PIII-3 characterized by pattern VEPs abnormalities limited to low voltage and delayed latency of P100 wave at 15’ spatial frequency and ERG abnormalities limited to low voltage 30 Hz retinal response without abnormalities of photopic 1 Hz and scotopic ERG. All patients showed cone ERG amplitude reduction followed by scotopic responses deterioration consistent with cone-rod ERG pattern with variable timing and progression rate. Pattern VEPs available for patients PII-1, PIII-1, PIII-2, PIII-3 were extinguished before ERG, while Flash VEPs became abnormal later in time showing prolonged latencies and progressive amplitude reduction During follow up all patients had progressive deterioration of both cone and rod ERG and VEPs responses except for PIII-2 and S2 who maintained an overall stability of photopic and scotopic ERG. MfERG performed on few occasions on S1 and S2 showed decreased central retinal function. At the last follow up, PII-1, PIII-1, PIII-3 had non-recordable ERG, pattern and flash VEPs; extinction of ERG occurred respectively at the age of 42, 21, 12 years.

PIII-4 (CAG > 170) showed at the age of 22 months an early involvement of both cone and rod responses. Follow up ERGs were not performed because of the severity of the clinical manifestation.

### Fundoscopic Examination and Tomographic Macular Analysis

At symptoms onset fundus oculi examination was normal in all except patient PII-1 and S2, for whom data were not available. Retinal changes occurred after the onset of visual symptoms but before ataxia in patients having visual onset first (PII-1, PIII-1, PIII-2, PIII-3, S1). Ophthalmic signs occurred after both neurological and visual onset in S2 and PIII-4. Early common findings ranged from subtle RPE changes in the macula to early signs of foveal atrophy. Over time PII-1, PIII-1, PIII-2, PIII-3 developed pale optic disk, attenuated retinal blood vessels, RPE changes from mid-periphery to periphery and extensive macular atrophy while S1 and S2 reported discreet pigmentary changes in the fovea followed by focal RPE hyperpigmentation.

At riluzole treatment baseline, all patients showed pigmentary changes in the fovea associated or not to optic nerve involvement. Macular OCT analysis showed no detectable outer layers of the retina, variable degree of retinal and foveal thinning according to retinal dystrophy stage and RPE changes. At the last follow up OCT, fundus examination and fundus photography showed a continuous worsening of macular atrophy documented by decreasing values of central retinal thickness.

### Ataxia and Neurological Impairment

Neurological onset preceded visual onset only in PIII-4 (CAG > 170) who presented at the age of 16 months with psychomotor regression. In the remaining patients presenting with visual impairment, the neurological examination at onset was normal, and SARA score was equal to 0. During follow up, the neurological examination, available before ataxia onset in PIII-1, PIII-2, PIII-3, showed as the first sign brisk lower limbs tendon reflexes, in the absence of Babinsky sign.

**Fig. 3:**
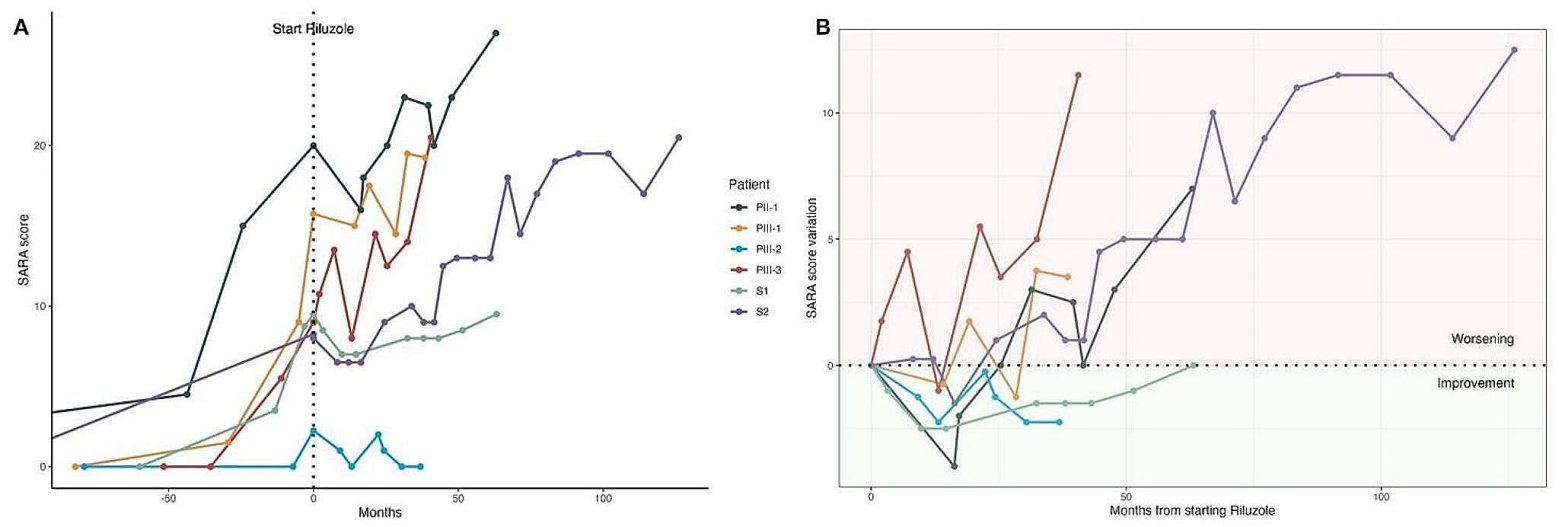
**A**: SARA Scores trend before and after riluzole treatment equalized at therapy onset (dotted vertical line); **B**: SARA Scores variation trend during riluzole treatment

SARA scores were progressively higher from onset to beginning of riluzole treatment in PII-1, PIII-1, PIII-3, S1 and S2, while PIII-2 ranged from 0 to 2.25. Ataxia showed up before treatment in all except PIII-2 at respectively 35, 17, 10.5, 66, 48 years in PII-1, PIII-1, PIII-3, S1, S2. Figure 3 summarizes SARA scores trend before and after riluzole treatment in all alive patients. At the last follow-up SARA scores ranged from 0 to 27 showing progressive neurological impairment in all except PIII-2.

### Visual Function and Ataxia Changes on Riluzole Therapy

Riluzole treatment was used by all but one patient (PIII-4); it was started at a mean age of 36.4 years (range 11.5–68) and lasted for a mean of 4.8 years (range 3.5-9). Figure 2 details the follow-up of the visual function pre- and post-riluzole treatment. It showed no effect on visual function in patients PIII-1 and PIII-3. Improvements occurred in PII-1, PIII-2, S1 and S2. In these four cases a stability in visual function was observed up to 5 years (and even more in S1 and S2) after the start of the treatment. Anatomical changes documented by imaging and optical tomography showed progressive pathological signs while visual electrophysiological data reported functional changes similar to visual function results: PII-1, PIII-1, PIII-3 showed progressive deterioration of cone and rod ERG and VEPs till extinction of both retinal and cortical responses. PIII-2 maintained over time residual rod-cone function and recordable but abnormal flash VEPs.

**Fig. 4:**
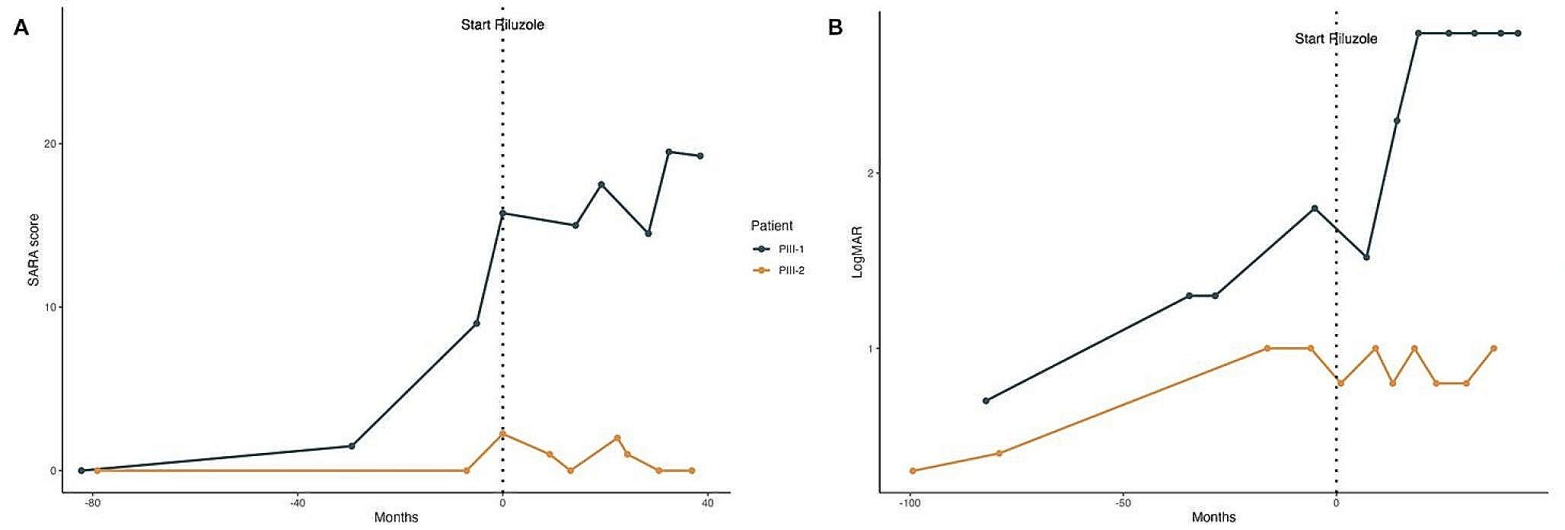
**A**: Longitudinal SARA score trend before and after riluzole therapy in patient PIII-1 and PIII-3. **B**: Longitudinal Visual acuity trend before and after riluzole therapy in patient PIII-1 and PIII-3

Figure 3 details the neurological follow-up. It shows that PIII-2 had an overall stabilization of visual deterioration without neurological impairment (no ataxia occurred until the last visit after 3.5 years of follow-up). He was the only one treated before ataxia onset, and, though he had the same triplet number as PIII-1, his disease course was apparently impacted by the start of the drug (Fig. 4). PIII-1 and PIII-3 showed a less steep deterioration trend after treatment compared to pre-treatment, during the first 2.5 years of therapy. PII-1 showed soon after therapy an improvement of the SARA score, as well as an overall stability lasting 3.5 years and followed by ataxia worsening. S1 and S2 showed an improvement of SARA scores soon after therapy, and an overall stability lasting respectively 5 and 3 years.

**Fig. 5:**
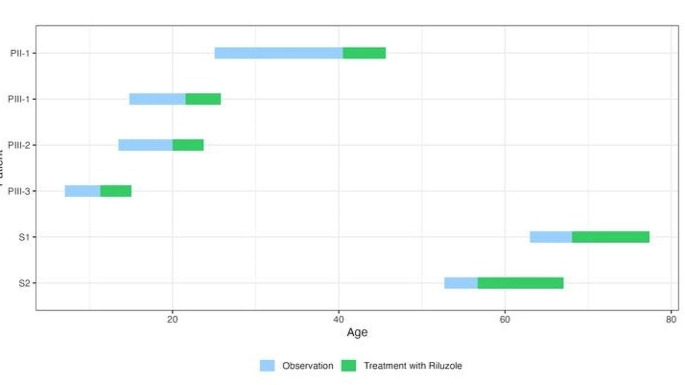
Timeline of observation during longitudinal follow-up before and after riluzole treatment

The timeline of the riluzole use is showed in Fig. 5, it depicts the long follow-up of the study. During the observation period, the electrocardiogram and laboratory profile (cell blood count, liver enzymes including AST, ALT, GGT, bilirubin and creatinine), performed every three months were always within the normal limits. No adverse event was registered.

## Discussion

This study represents a possible advancement toward a better management of an ultra-rare disease such as SCA7. First, it reports a detailed description of the ophthalmologic profile of these patients that in part confirms and even introduces new concepts on the peculiarity of visual impairment in SCA7. Second, the follow-up results inform on the longest use of riluzole in cerebellar ataxia (CA), and a very long administration of riluzole in a neurodegenerative condition (in fact patients with amyotrophic lateral sclerosis, for whom the drug is currently indicated, tend to have shorter follow-up because of the poor prognosis and the trend to withdraw the drug in the absence of effect). These results confirm a substantial safety profile of the drug and suggest possible beneficial effects in SCA7.

Concerning the visual involvement in our patients, visual acuity was the most affected function with earlier onset, followed by color sensitivity, contrast sensitivity and concomitant central visual field restriction. The literature reports early visual acuity involvement even if asymptomatic impairment of color vision in the blue – yellow axis has been suggested to occur years before patients report visual failure [24]. Etiology of visual loss was a retinal disorder as confirmed by the combination of clinical clues with visual electrophysiology showing early 30 Hz flicker ERG and pattern reversal VEPs abnormalities. These are consistent with macular loss of function. Rod-ERG involvement occurred over time suggesting a clinical phenotype of cone-rod dystrophy [25]. These results are in line with studies in adult patients based on full-field ERG, multifocal ERG, microperimetry, fundus oculi examination and OCT [24, 26]. The progression of visual impairment was initially slow in our patients, followed by rapid deterioration evolving toward complete blindness. Tests of function were abnormal before anatomical signs at eye imaging and examination, such as vascular attenuation, optic disc pallor, and peripheral pigmentary changes at fundus oculi inspection [6, 24, 26]. Patients tend to look for medical advice later when visual acuity is already very low [27]. Taken together, these data suggest that retinal photoreceptors may undergo dysfunction before degeneration and the importance of early diagnosis when an initial therapeutic window may still allow compensatory mechanisms to be effective.

Studies of retinal function and histology in animal models overexpressing mutant ataxin-7 support the notion that visual failure in SCA7 results from early dysfunction rather than demise of photoreceptors [28, 29]. Likely, ataxia might result primarily from neuronal dysfunction followed by neuronal demise in Purkinje cells of cerebellum [30]. Research on mechanisms of the retinal disorder in SCA7 showed that overexpression of human ataxin-7 provokes ERG abnormalities and outer retinal degeneration [28, 29]. Ataxin-7 interacts with CRX, a transcription factor regulating photoreceptor genes [31, 32] known to cause cone-rod dystrophy [24, 26].

The natural history of our patients shows, in agreement with the literature [4, 8, 9, 33] that larger CAG repeats correlate with earlier onset age, faster rate of progression and shorter disease duration, with anticipation in the offspring. Vision impairment was the initial manifestation of the disease in all but one of our patients, having CAG repeats ranging between 39 and 63. The exception was the youngest patient with onset at 16th months, higher number of CAG repeats (> 170) and more rapid progression until death at 4 years of age. This child belongs to infantile/early childhood onset subgroup based on initial clinical presentation, age of onset, and repeat expansion size [33]. She was reported to started with gait ataxia, in apparent contrast with David and coauthors who showed that longer CAG tracts lead increased chance of visual failure preceding cerebellar ataxia (CA) [8]. However, due to the very young age of our patient, an initial mild visual loss could have been undetected for the inability to express visual impairment, compared to the self-evident motor impairment [34].

The results of this study suggest that riluzole treatment may have a beneficial symptomatic effect extended to 3–5 years when used after disease onset (see Figs. 2b and 3b). The same treatment before ataxia onset may even have a disease-modifying effect, accounting for different outcomes between sibling with the same number of CAG repeats (see curves of PII-1 and PII-2 in Fig. 4). In the hypothesis that a dysfunction of retinal photoreceptors precede degeneration it is expected that early treatment can have e better efficacy. This couple of brothers followed in Italy, and in part the American sisters, as well as the overall dynamics of the riluzole effects on disease progression, seem to suggest that there is a therapeutic window to start this therapy in an early phase of SCA7, when only paraclinical ophthalmologic data show abnormalities, without clinical symptoms (stage 1 according to Horton [35]), and the treatment may have modifying consequences on the outcomes.

One of the limitations of this study is the fact that riluzole was given at different stages of the disease. Therefore, it cannot be excluded that the stabilising effect observed in the early stages could be due to the natural history of the disease. Literature suggests that SCA7 is a truly progressive disease. However, further research is needed to clarify this issue. Another limitation of this study was that it was designed to study a specific type of degenerative cerebellar disease. However, this approach was limited by the relatively small number of patients affected by this ultra-rare disease.

A randomized, controlled trial with a delayed-start study design (NCT03660917) is ongoing to confirm a symptomatic effect of riluzole in this form of SCA, as well as to support the possibility that this drug may represent a possible disease-modifying approach.

## Data Availability

The data that support the findings of this study are not openly available due to reasons of sensitivity and are available from the corresponding author upon reasonable request.
