# Correction: Long-Term Follow-Up Before and During Riluzole Treatment in Six Patients from Two Families with Spinocerebellar Ataxia Type 7

**DOI:** 10.1007/s12311-024-01742-6

**Published:** 2024-09-13

**Authors:** Agnese Suppiej, Chiara Ceccato, Radouil Tzekov, Iveta Cermakova, Francesco Parmeggiani, Gianmarco Bellucci, Marco Salvetti, Theresa Zesiewicz, Giovanni Ristori, Silvia Romano

**Affiliations:** 1 Department of Medical Sciences, University of Ferrara, Ferrara, Italy; 2 Robert Hollman Foundation, Padova, Italy; 3 ERN-EYE Network - Center for Retinitis Pigmentosa of Veneto Region, Camposampiero Hospital, Camposampiero (Padova), Italy; 4 Department of Ophthalmology, University of South Florida, Tampa, FL USA; 5 Department of Translational Medicine for Romagna, University of Ferrara, Ferrara, Italy; 6 Center for Experimental Neurological Therapies, Department of Neurosciences, Mental Health and Sensory Organs (NESMOS), Faculty of Medicine and Psychology, Sant’Andrea Hospital, Sapienza University of Rome, Rome, Italy; 7 IRCCS Istituto Neurologico Mediterraneo (INM) Neuromed (M.S.), Pozzilli, IS Italy; 8 Department of Neurology, University of South Florida, Tampa, FL USA; 9 Neuroimmunology Unit, Fondazione Santa Lucia, Rome, Italy

**The Cerebellum**

10.1007/s12311-024-01714-w

In the original version of this article, the given and family names of authors were incorrectly structured. The name was displayed incorrectly in all versions at the time of publication.

Correct list of authors as it should appear at proofs are as follows:

Agnese Suppiej, Chiara Ceccato, Radouil Tzekov, Iveta Cermakova, Francesco Parmeggiani, Gianmarco Bellucci, Marco Salvetti, Theresa Zesiewicz, Giovanni Ristori, Silvia Romano

The original article has been corrected.

